# Genome-Wide Linkage Analysis of Malaria Infection Intensity and Mild Disease

**DOI:** 10.1371/journal.pgen.0030048

**Published:** 2007-03-23

**Authors:** Christian Timmann, Jennifer A Evans, Inke R König, André Kleensang, Franz Rüschendorf, Julia Lenzen, Jürgen Sievertsen, Christian Becker, Yeetey Enuameh, Kingsley Osei Kwakye, Ernest Opoku, Edmund N. L Browne, Andreas Ziegler, Peter Nürnberg, Rolf D Horstmann

**Affiliations:** 1 Department of Molecular Medicine, Bernhard Nocht Institute for Tropical Medicine, Hamburg, Germany; 2 Institute of Medical Biometry and Statistics, University Hospital Schleswig-Holstein, Campus Lübeck, University at Lübeck, Lübeck, Germany; 3 Kumasi Centre for Collaborative Research in Tropical Medicine, Kumasi, Ghana; 4 Max Delbrück Centre for Molecular Medicine, Berlin, Germany; 5 Cologne Center for Genomics, University of Cologne, Cologne, Germany; 6 Department of Community Health, Kwame Nkrumah University of Science and Technology, Kumasi, Ghana; University of Oxford, United Kingdom

## Abstract

Although balancing selection with the sickle-cell trait and other red blood cell disorders has emphasized the interaction between malaria and human genetics, no systematic approach has so far been undertaken towards a comprehensive search for human genome variants influencing malaria. By screening 2,551 families in rural Ghana, West Africa, 108 nuclear families were identified who were exposed to hyperendemic malaria transmission and were homozygous wild-type for the established malaria resistance factors of hemoglobin (Hb)S, HbC, alpha^+^ thalassemia, and glucose-6-phosphate-dehydrogenase deficiency. Of these families, 392 siblings aged 0.5–11 y were characterized for malaria susceptibility by closely monitoring parasite counts, malaria fever episodes, and anemia over 8 mo. An autosome-wide linkage analysis based on 10,000 single-nucleotide polymorphisms was conducted in 68 selected families including 241 siblings forming 330 sib pairs. Several regions were identified which showed evidence for linkage to the parasitological and clinical phenotypes studied, among them a prominent signal on Chromosome 10p15 obtained with malaria fever episodes (asymptotic *z* score = 4.37, empirical *p*-value = 4.0 × 10^−5^, locus-specific heritability of 37.7%; 95% confidence interval, 15.7%–59.7%). The identification of genetic variants underlying the linkage signals may reveal as yet unrecognized pathways influencing human resistance to malaria.

## Author Summary

In tropical Africa, virtually all children become infected with malaria parasites. Most of them experience several malaria attacks per year, and over a million die from disease complications. Sickle-cell anemia, thalassemias, and other inherited red blood cell disorders indicate that malaria has selected for human genetic variants, but no attempts have so far been reported to systematically screen the human genome for malaria-resistance factors. We describe a genome-wide linkage analysis performed in children living in rural Ghana, West Africa, including approaches to select an informative study cohort and to assess, over a period of 8 mo, individual disposition to malaria parasitemia, fever episodes, and anemia. Families carrying the known malaria-protective red blood cell disorders were excluded, infection intensities were adjusted to the use of mosquito-protection devices, and parasitological and clinical findings were corrected according to the state of partial malaria immunity, which, under constant exposure, gradually develops over the first 10 y of life. The study revealed several genomic regions showing evidence for linkage to the various malaria phenotypes recorded, among them a prominent signal on Chromosome 10 correlated to the frequency of fever episodes. Future identification of genes involved is expected to reveal previously unrecognized pathways that may protect children against malaria.

## Introduction

Malaria caused by Plasmodium falciparum is one of the leading causes of human morbidity and mortality worldwide, predominantly affecting populations of resource-poor countries in the south [1]. Drawbacks in developing effective control measures have stressed the demand for research aiming at a better understanding of basic elements of parasite biology and disease pathology.

The blood stages of the parasite comprise asexual forms, which maintain the infection and cause disease, and sexual forms, which transmit the infection [2]. Asexual blood parasite counts are the established measure of infection intensity [3], whereby reports on substantial variations over a short period of time indicated that many measurements may be required for appropriate estimates [4].

Clinically, malaria presents as a mild form of acute febrile episodes and anemia, or as a severe form, which comprises a complex syndrome of life-threatening complications [5]. While the severe form causes an enormous humanitarian burden, it does not affect more than 1%–2% of the residents of endemic areas [6], whereas the mild form predominates in terms of quantitative morbidity and economic reasoning [1,7,8]. While the non-specific symptoms of fever, headache, and nausea make the diagnosis of malaria fever episodes difficult to ascertain, a simple case definition proposed by the World Health Organization (WHO) based on fever and parasitemia is generally accepted due to its high sensitivity and specificity in endemic areas, where the vast majority of such episodes are in fact caused by malaria [9].

A second clinical feature of mild malaria is anemia. It affects an enormous number of children in endemic areas [10] and may present as a chronic, subacute, or acute, sometimes life-threatening form [5]. Its pathogenesis is considered multifactorial and may include the destruction of infected and uninfected erythrocytes and bone-marrow dysfunction, whereby the relative contributions of these factors and their roles in the various forms of malarial anemia have not yet been resolved [11].

The effect of human genetics on malaria has long been recognized when the theory of balancing selection was substantiated for thalassemias, sickle-cell anemia, and other red blood cell disorders [12]. Twin studies and heritability estimates have subsequently confirmed the influence of host genetics, which was shown to be most pronounced in children [13–15]. Candidate gene approaches have indicated a number of additional variants to be involved including those of the major histocompatibility complex and a cytokine-gene cluster on Chromosome 5q31-q33 [16]. However, no systematic analysis has been reported to address human genetics in malaria more comprehensively.

Here we report on an autosome-wide linkage analysis for P. falciparum infection intensity and mild clinical malaria among African children selected not to carry any of the classic malaria resistance genes. As markers, 10,000 single-nucleotide polymorphisms (SNPs) were used.

## Results

### Phenotypes

**Figure 1:**
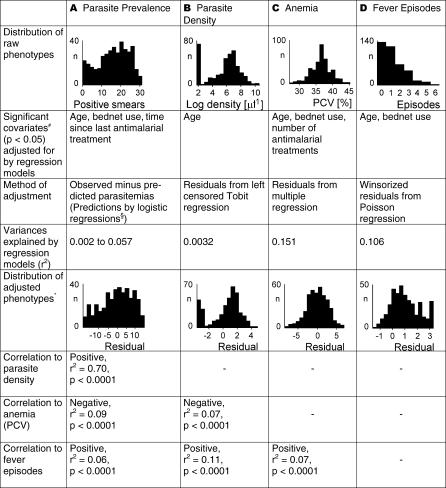
Phenotype Adjustments of the Study Group (392 Siblings) (A) Presence of asexual blood forms of P. falciparum in 31 weekly blood smears. (B) Log of the 75th percentile of 31 parasite density values per individual. (C) Median of 16 PCV assessments per individual. (D) Malaria fever episodes were defined following WHO recommendations whereby multiple malaria attacks within 3 wk were considered recrudescences and counted as one episode (see Materials and Methods). ^#^Gender did not significantly influence any of the phenotypes. For the phenotype of parasite density, the effects of antimalarial treatments were addressed by exclusion of density values following 3 wk after treatment (see Materials and Methods). The phenotype of fever episodes was not corrected for the number of antimalarial treatments because of the direct causal relationship between disease episodes and treatments. ^§^31 weekly regression models. ^*^Anemia (PCV) was normally distributed, Shapiro-Wilk W test, *p* > 0.2.

392 siblings of 108 families resident in West Africa were followed over a period of 31 wk, which covered an entire rainy season. Prevalences of P. falciparum blood trophozoites, parasite densities, and interim or present malaria fever episodes were monitored weekly and anemia as indicated by the packed blood-cell volume (PCV) was determined biweekly. Compliance was as follows; 98.8% of 12,152 parasitemia assessments, 95.4% of 6,272 PCV assessments, and 98.5% of 12,152 assessments for malaria fever episodes were recorded with a maximum of data missing per planned visits of single participants of 13/31, 6/16, and 18/31 records, respectively. Results from regression models for analyzing the effect of age, bednet use, and intake of antimalarials on the various phenotypes are summarized in Figure 1. Gender had no significant effect on any of the phenotypes, and therefore was not included in the final regression models used for phenotype corrections.

### Genotyping

Based on a ranking that favored high levels of parasite densities in conjunction with high intrafamilial variability (see Materials and Methods), 377 individuals of 68 families were selected for genotyping, including 136 parental individuals and 241 siblings, who formed 330 sib-pairs. Applying the Affymetrix Human Mapping 10K array yielded an overall autosomal calling fraction of 94.5% for the raw genotypes. These were defined as SNPs for which definitive genotypes were obtained. After application of the quality control procedure, 1,524 autosomal markers (15.2%) were excluded from further analysis. The remaining markers yielded a mean information content of 0.976 (SD ± 0.029, range 0.510–1.000).

### Linkage Analysis

**Figure 2:**
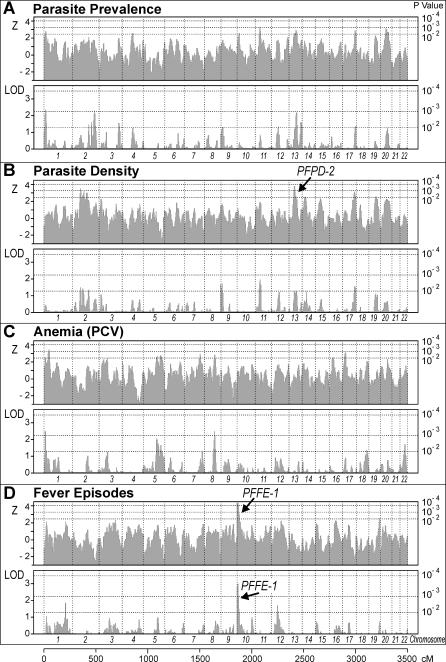
Quantitative-Trait, Multipoint Linkage Analysis of the Key Phenotypes of Uncomplicated Human Malaria (A and B) Parasitological phenotypes. (C and D) Clinical phenotypes. A 10k-Affymetrix SNP array was used for genotyping 377 individuals comprising 241 siblings of 68 families. Results obtained by NPL are presented in the upper panels (z scores [Z] on the left axis) and those of HE are presented in the lower panels (LOD scores [LOD] on the left axis). Empirical significance levels (*p*-values indicated by dotted lines) were estimated by 100,000-fold Monte-Carlo permutations.

The nonparametric linkage analysis (NPL) and Haseman-Elston multipoint linkage analysis (HE) were applied (Figure 2A–2D). Parasite prevalence, parasite density, fever episodes, and anemia were analyzed as quantitative phenotypes. The most prominent result was a linkage signal for malaria fever episodes on Chromosome 10p15.3–10p14, which reached statistical significance in both the NPL and HE analyses. NPL showed an asymptotic *z* score of 4.37 (empirical *p*-value = 4.0 × 10^−5^) between SNP markers rs952153 and rs1964428 marking the interval of 5.9–12.0 cM and 2.5–3.5 Mb of the genetic and physical chromosomal maps, respectively (Figure 2D). HE showed a maximum asymptotic logarithm of odds (LOD) score of 3.03 (empirical *p-*value = 2.1 × 10^−4^) at marker rs1964428 corresponding to 12.0 cM/3.5 Mb (Figure 2D). The locus-specific heritability was estimated to be 37.7% (95% confidence interval, 15.7%– 59.7%) at 11.2 cM. The linkage region was termed *PFFE-1* for P. falciparum-fever episode 1. The signal was robust to variations in data analysis, including the use of a raw phenotype without adjustments for covariates (*z* score of 4.52), or the use of a wider definition of malaria fever episodes that included afebrile malaria episodes diagnosed by the study physicians (*z* score of 4.04). The 2.2 *z* score support interval (corresponding to a 1-LOD support) encompassed a 27.4 cM/11.0 Mb distance containing 71 annotated or hypothetical genes. Functional candidates include genes encoding a platelet-type phosphofructokinase *(PFKP)* also expressed in red blood cells, an inducible 6-phosphofructo-2-kinase/fructose-2,6-bisphosphatase *(iPFK-2/PFKFB3),* the alpha chain of the interleukin-15 receptor *(IL15RA),* the alpha chain of the interleukin 2 receptor *(IL2RA),* protein kinase C theta *(PRKCQ),* the GATA-binding protein 3 *(GATA3),* and a gene similar to that of the interleukin 9 receptor precursor *(LOC439945).*

A further region with evidence for linkage was found using parasite density as the phenotype. The NPL analysis yielded a signal on Chromosome 13q with a maximum asymptotic *z* score of 3.73 (empirical *p*-value = 2.3 × 10^−4^) between rs2147363 at chromosomal position 55.0 cM/51.4 Mb and rs726540 at 55.5 cM/52.3 Mb (Figure 2B). HE resulted in a LOD score of 1.19 at this position (Figure 2B). The locus-specific heritability was estimated to be 33.7% (95% confidence interval, 9.8%–57.6%). The region was termed *PFPD-2* for P. falciparum-parasite density 2, whereby another linkage region with parasite density had previously been reported [17–19]. The 2.2-*z* support interval of *PFPD-2* encompassed 24.2 cM/32.4 Mb containing 158 annotated or hypothetical genes. Possible functional candidates include genes encoding the lymphocyte cytosolic protein 1 *(LCP1),* S-formylglutathione hydrolase (esterase D, *ESD*), the cysteinyl leukotriene receptor 2 *(CYSLTR2),* and the endothelin receptor, nonselective type, *(EDNRB).*

Furthermore, a signal on Chromosome 1p36 at 18 cM/9 Mb provided evidence for linkage with both parasite prevalence (LOD score of 2.31; empirical *p*-value = 5.3 × 10^−4^), and PCV (LOD score of 2.45; empirical *p*-value = 3.9 × 10^−4^) at adjacent marker positions (rs205474 and rs966134, respectively; Figure 2A and 2C). The NPL *z* scores were low in both instances (2.75 and 2.36, respectively).

Finally, no evidence was obtained for linkage of parasite density or malaria fever episodes to 5q31-q33 and to the MHC region on 6q23, respectively, which had previously been reported. In the present study, weak evidence was obtained that malarial anemia might be linked to 5q31-q33 (*z* score = 2.7, LOD score = 1.8) (Figure 2C).

## Discussion

To our knowledge, this is the first genome-wide approach to identify human genetic variants influencing susceptibility and resistance to malaria. Since the seminal observations on balancing selection with inborn red blood cell disorders, malaria is a prominent element in human genetics. The importance of the classic malaria-protective red blood cell traits is in the present study highlighted by the large proportion of 86% of families found to be affected in the initial survey of our study population. These were excluded from the study in order to concentrate the search on as yet unrecognized human genetic variants [16,20]. As genetic influences were reported to be of particular relevance in childhood malaria [14], we limited our study to children aged 0.5–11 y.

Assessing the phenotype of malaria infection intensity remains a challenge because it is uncertain to which extent any limited number of parasite counts truly reflect the infection intensity [4]. In addition, infection intensities may strongly depend on exposure, which is a variable difficult to assess in field studies. In the present study, the use of bednets and window screens to reduce exposure by preventing mosquito bites was addressed by data adjustments and exclusions of families, respectively (see Materials and Methods). It may be considered an advantage that the NPL and HE methods applied are based on intra-familial evaluations because malaria exposure is likely to be homogeneous within families living in the same households.

As expected, antimalarial treatments had an effect on all phenotypes studied. The influence on parasite prevalences and parasite densities was found to be limited to the two subsequent assessments, therefore it was addressed by correcting the respective values of prevalences and by excluding the corresponding densities (see Materials and Methods). In contrast, the influence on anemia was corrected for by adjusting the overall phenotype because epidemiological observations on the effect of drug resistance on anemia suggest possible long-term effects [22,23]. Concerning the number of fever episodes, no adjustments were made because they might have neutralized the essential phenotypic information due to the direct causal relationship between disease episodes and treatments.

Of all covariates tested, age had the strongest effect and was included in all phenotype adjustments. In children older than 6 mo as in the present cohort, the age effect on malaria in endemic areas is dominated by the gradual development of a certain degree of adaptive immunity, termed semi-immunity. This is reflected by a successive decrease over age of the number of fever episodes, the degree of anemia, parasite densities, and, at relatively high age, parasite prevalences [24–27]. Therefore, the phenotypes addressed may be influenced by both innate resistance and adaptive immunity, whereby innate resistance may have a predominant influence in younger children and adaptive immunity in older ones. This may focus the linkage signals obtained in this study on variants that are relevant under both conditions.

The phenotypes studied showed significant correlations between each other. This is in agreement with the general understanding that all signs and symptoms of malaria result from parasitemia. The explained variances in most instances were low, however, leaving room for separate genetic influences. As expected, the correlation between parasite prevalences and parasite densities was exceptionally high. Despite this, both were included as separate phenotypes because there is evidence to suggest that they are under distinct genetic influences. First, epidemiological findings including those of the present study (unpublished data) indicate that semi-immunity suppresses high parasite densities significantly more efficiently than low parasite densities [24], which suggest distinct elements of adaptive immunity. More importantly, HbS has been shown to protect from high parasite density but not from parasitemia itself [28], indicating that mechanisms of genetic resistance may affect high parasite density specifically.

Evaluation of the data using established linkage methods revealed several prominent linkage signals. Interestingly, locus-specific heritability calculations performed for two of these linkage regions indicated that, in both cases, approximately 35% of the total phenotype variability was attributable to these loci in families who did not carry any of the established malaria resistance factors. These estimates allow us to postulate the effect of a major locus in both instances, which would support a recent conclusion that susceptibility and resistance to infectious diseases may be governed by single major genes rather than by a large number of genes each exerting a small influence [29].

The region showing strongest and significant linkage concerned the phenotype of malaria fever episodes *(PFFE-1).* Notably, the signal was found in both model-free approaches. Furthermore, it was robust to variations in phenotype definitions, which may be of particular importance because the non-specific symptoms of malaria fever episodes make the clinical diagnosis uncertain. That we found the strongest linkage signal with this particular phenotype may relate to the fact that fever regulation might be similar regardless of whether it is influenced by innate resistance or adaptive immunity, with respect to the age-dependent bias introduced into our study cohort by these two factors, as described above. The underlying genetic variant may be of more general interest because it may relate to the regulation of the systemic inflammatory response.

A number of additional regions with evidence for linkage were identified which did not reach statistical significance. Therefore, they are not discussed in any detail, although experiences in other complex diseases have shown that weaker linkage signals may as well lead to the identification of relevant genetic variants [30]. The linkage regions described comprise a number of genes which may be classified as functional candidates because their products are operative in immune regulation or red blood cell metabolism. However, regarding their established functions, we consider none of them a prime candidate.

No support for our data can be derived from previous linkage studies in mouse malaria. Studying parasite density in murine Plasmodium chabaudi infection, evidence has been obtained for linkage regions on Chromosomes 3, 5, 9, 11, and 17 [31] but not on Chromosome 14, which covers the synteny of the linkage region on human 13q we obtained for *P. falciparum-* parasite density (NCBI, http://www.ncbi.nlm.nih.gov/Homology/). This is not unexpected because P. falciparum substantially differs from *P. chabaudi* in that P. falciparum-infected red blood cells adhere to the vascular endothelium [2], which may have a strong influence on parasite biology. Further linkage studies on murine malaria are limited to the phenotype of cerebral manifestations in *Plasmodium berghei* infections [31], which cannot be compared to our clinical phenotypes of uncomplicated malaria, and identified regions on Chromosomes 1, 11, and 17 but not on 13 and 2, which cover the regions syntenic to *PFFE-1* on 10p.

To our knowledge, this is the first time that the Affymetrix HMA10k chip was used for genotyping individuals of African descent. The raw genotypes yielded a call rate of 94.5%, which nearly reached 95% considered sufficient for optimal assay performance [32] and was comparable to 96.9% reported for Caucasians [33]. This provides a basis for using the chip in African populations.

## Materials and Methods

### Participants*.*

The study was conducted in the Asante Akim North District, Ashanti Region of Ghana, West Africa, a region classified as hyperendemic for malaria by a cross-sectional prevalence of 0.54 for P. falciparum.

Ethical approval was obtained from the Committee for Research, Publications and Ethics of the School of Medical Sciences, Kwame Nkrumah University of Science and Technology, Kumasi, Ghana. All procedures were explained in the local language, and consent was obtained from both parents. Parents of 2,551 families were recruited who had three or more children below the age of 12 y and agreed to participate. Venous blood samples of 2 ml were obtained from both parents and preserved by addition of an equal volume of 8 M urea. The genetic variants of hemoglobin (Hb)S, HbC, alpha^+^ thalassemia deletion 3.7, and glucose-6-phosphate-dehydrogenase (G6PD) deficiency A-, which were considered to possibly influence susceptibility to P. falciparum parasitemia and mild malaria, were determined, and 346 (13.6%) families were identified not segregating any of the traits.

Of the 346 families, a study group of 392 siblings of 108 families was selected based on (i) the logistic criterion that their homes clustered in 16 of the 30 villages included in the initial survey and (ii) that they did not live in homes equipped with window screens, which a posteriori were found to significantly reduce parasite prevalences from 0.54 to 0.35 (*p* < 0.001) and other parameters of malaria infection intensity. All families belonged to the ethnic group of Akan.

A subset of 377 members of 68 families were selected from the study group by excluding siblings who were absent at more than two assessments and by a ranking that favored high levels of parasite densities of P. falciparum in conjunction with high intrafamilial variability. The ranking score was based on the product of the mean of log parasite densities of P. falciparum within sib-ships multiplied by the standard deviation of log parasite densities within sibships. Families with highest scores were selected until 377 individuals were identified for genotyping. The genetic study group comprised 136 parents with 241 children, 52.5% boys and 47.5% girls, who, with a mean of 3.54 siblings per family, formed 330 sib pairs. Their median age was 5 y (range 0.5–11 y; IQR 3–8 y).

### Phenotype assessments.

The children were phenotyped from May 20 to December 20, 2002. Weekly assessments by the visit of a trained physician included a medical history, measurement of body temperature by an infrared ear thermometer, a blood sample by finger prick or heel prick (approximately 100 μl), and in case of disease symptoms, a physical examination. The installation of window screens in homes and the use of bed-nets were recorded.

Weekly malaria smears were prepared at the study site, and in the laboratory they were stained with Giemsa and examined [34]. Parasite species were assessed, and parasite counts were recorded per 200 leukocytes (if >10 parasites/200 leukocytes) or 500 leukocytes (if ≤10 parasites/200 leukocytes) by two independent examiners. Parasite densities were calculated assuming a leukocyte count of 8,000/μl [34]. If the densities as determined in the two counts differed by a factor of three or more, a third independent count was obtained. The median parasite density of two or three counts was included in the analysis.

Weekly point prevalences of malaria parasitemias showed a median of 54% (range 40%–61%) yielding a cumulative period prevalence of 99%. Contributing 98% of all parasitemias, P. falciparum was the predominant species; Plasmodium ovale or Plasmodium malariae were found in 19% and in 90% of these in combination with P. falciparum. For the assessments of parasite prevalences and parasite densities, only P. falciparum and only the disease-causing asexual forms were included, which showed a median point prevalence of 53.1% and a period prevalence of 99%. Parasite densities of P. falciparum ranged from 0–317,360 parasites per μl, with an overall median of 32 and a 75th percentile of 680 in 12,011 assessments.

Anemia was on the spot assessed as PCV by capillary hematocrit centrifugation using 70 μl EDTA anticoagulated capillary tubes (Becton Dickinson, Germany) and mobile centrifuges. To reduce iron-deficiency as a possible confounder, prior to phenotyping, all children were treated against hookworm infection with 400 mg albendazole followed by oral iron supplementation of 2 mg/kg Fe^2+^ over 6 wk. The median PCV value of 16 biweekly assessments was used as the phenotype in all cases because none of the children showed evidence for any other disease causing substantial anemia. As determined by 16 biweekly PCV measurements, mild anemia defined by a PCV of <33% in the age group of 0.5–6 y and of <36% in the age group of 6–14 y [11] was found at 1,625 of 5,986 assessments (27%) affecting 326 (66%) of the children.

Mild malaria attacks were defined following WHO recommendations [9], first, by either assessing fever by a tympanic temperature of >37.7 °C, corresponding to a rectal temperature of >37.6 °C [35], or reported fever within the previous 4 d and second, a blood smear positive for asexual forms of P. falciparum at any density. The number of fever episodes during the observation period of 31 wk was counted for each individual, whereby multiple episodes within 3 wk were considered recrudescences and counted as one episode [36].

Applying this definition resulted in the identification of 504 malaria fever episodes affecting 257 children, which corresponds to a period prevalence of 66%. In addition, 34 febrile states of other etiologies including upper respiratory tract infections, lower respiratory tract infections, pneumonia, measles, and urinary tract infections were diagnosed by the study physicians. Suspected malaria attacks were treated by a standard dose of chloroquine or amodiaquine following national guidelines, other illnesses as deemed appropriate.

In greater detail, a tympanic temperature of >37.7 °C was measured at 365 visits. Subsequent malaria smears showed that 248 (68%) of the fever attacks occurred in conjunction with P. falciparum parasitemias (and four with other malaria parasites). Fever attacks reported by parents or guardians to have occurred between two examinations were recorded at 870 visits, whereby blood smears at the time of examination were positive for P. falciparum at 522 of these instances, and 133 of the attacks had reportedly been treated with antimalarials. The study physicians at 587 instances suspected malaria attacks at the time of examination, 389 (66%) of which were retrospectively supported by the presence of P. falciparum parasitemia. Of 631 mild malaria attacks as defined by WHO recommendations, 57% were in agreement with malaria diagnoses made by the study physicians, whereas 3% were classified by the study physicians as a febrile illness other than malaria.

### Phenotype definitions and adjustments.

Regression models were used for adjusting the raw phenotypes for influences of covariates (Figure 1). Continuous covariates were modeled using multivariable fractional polynomials [37,38] at the nominal 5% test level. The choice of the 5% test level has been discussed in [38]. Specifically, we looked for non-linearity by fitting a second-order fractional polynomial to the data. The best power transformation x^p^ of covariate x is found, with the power p chosen from −2, −1, −0.5, 0, +0.5, +1, +2, +3, where x^0^ denotes ln x. For example, for *p* = 0, 0.5 the model is b_0_ + b_1_ ln(x) + b_2_ √x. The set includes the straight line, i.e., no transformation *p* = 1, and the reciprocal, logarithmic, square root, square, and cubic transformations. Even though the set is small, the powers offer considerable flexibility. The test is performed by comparing the difference in model deviances with a χ^2^ distribution on 1 degree of freedom. The resulting *p*-value is approximate and is justified by statistical arguments [37]. Pearson residuals from logistic regression and ordinary residuals from linear and Tobit regressions [39] were used for further analyses.

The difference between the sum of observed weekly P. falciparum parasitemias (1 or 0) minus the sum of predicted probabilities for parasitemias of an individual were calculated and used as phenotype. The predictions were derived from the study population by logistic regression models for parasitemia for each week's set of data with respect to the influences of age, time period since the last use of antimalarials, and the use of bed-nets (Figure 1).

Parasite densities recorded within 2 wk following the administration of antimalarials were found to be significantly lower than parasite densities determined at other time points (Wilcoxon rank sums tests, *p* < 0.05) and were therefore excluded. Since the 31 weekly parasite-density values (or their log-transformations) of an individual deviated from a normal distribution, quantiles were used. The 75^th^ percentiles were chosen because they were substantially more informative than the median values, which would have been zero in nearly half the assessments. The 75^th^ percentiles were log-transformed, whereby half the detection threshold of 16 parasites per μl was added before taking the logarithm. The phenotype was adjusted to age by calculating residuals from a Tobit regression model, whereby 74 of 392 observations were left censored (Figure 1).

The median values of 16 biweekly PCV assessments were adjusted using a multiple regression model which included the variables of age, number of antimalarial treatments, and use of bednets. Age was modeled as a second-degree fractional polynomial with exponents of −2, 2, the number of antimalarial treatments were considered untransformed, and bednet use was coded as a dummy variable (Figure 1).

The numbers of malaria fever episodes of each individual were adjusted for age and bednet use by calculating residuals from a Poisson regression model. Age was modeled as a second-degree fractional polynomial with exponents of −0.5, −0.5, bednet use was coded as a dummy variable. To avoid outliers for HE, phenotypic values above the 95th percentile were winsorized [40] (Figure 1).

Data analysis showed that siblings of families using mosquito protection by window screens had significantly reduced parasite prevalences, parasite densities, and numbers of fever episodes. As this indicated a marked reduction in exposure, the siblings were excluded a posteriori. The use of bednets was associated with slightly increased parasite prevalences, anemia, and numbers of fever episodes. As bednet use was heterogeneous within one family, all phenotypes were corrected accordingly. Intuitively, the associations appear paradoxical but may be explained by hypothesizing that households living under particularly high exposure, which implies particular molestation by mosquito bites, might use bednets preferentially, albeit with insufficient effectiveness [21].

### Genotyping.

Venous blood samples of 2 ml were obtained at the end of the study period and preserved by the addition of urea. DNA was extracted by a standard procedure (NucleoMag 96 Blood, Macherey-Nagel, Germany). HbS, HbC [41], and alpha^+^ thalassemia [42] were determined as described. G6PD variants *A* (A376G), and *A-* (A376G, G202A) [43] were typed following a PCR-flourescence-resonance-energy-transfer hybridization method [41], whereby forward and reverse primers (F, R) as well as fluorescein (FL) or LC640red (LC640) (Roche, Germany) labeled anchor (A) and sensor (S) oligonucleotides were used: Variant A376G was typed with F: 5′-tgtgtgtctgtctgtccgtgt-3′, R: 5′-aacggcaagccttacatctg-3′, A: 5′-FL-cgatgatgcagcctcctaccagcgc-3′, S: 5′-LC640-tcaacagccacatggatgccctcc-3′ and variant G202A with F: 5′-cagctgccctgccctcag-3′, R: 5′-cttgaagaagggctcactctgtttg-3′, A: 5′-LC640-cagaaggccatcccggaacagcc-phosphate-3′, S: 5′-gcatagcccacgatgaaggtgttttc-FL-3′. Allele frequencies of HbS, HbC, alpha^+^ thalassemia, and G6PD *A-* were estimated to be 0.071, 0.058, 0.175, and 0.063, respectively. *G6PD* variant *A,* which was previously reported to show only a slightly decreased enzyme activity of 80% compared to the normal variant *B* (A376) [44], was retrospectively found not to show a significant influence on any of the phenotypes (Wilcoxon rank sums tests of genotypes AA or A, AB, and BB or B on the phenotypes parasite prevalence (*p* = 0.13), parasite density (*p* = 0.55), anemia (*p* = 0.36), and fever episodes (*p* = 0.80); therefore children being homozygous, heterozygous, or hemizygous for *G6PD B* and/or *A,* were included in the study group. Paternities and maternities were assessed by typing the short tandem repeat markers D1S2782, D6S273 (Genethon, www.genethon.fr), D5S816, D7S2212, D11S1984, D17S1299, and D17S1294 (The Cooperative Human Linkage Center, http://lpgws.nci.nih.gov/CHLC/), in(TG)_n_ of CD36 [45], and IL10G [46], all showing heterozygosity of ≥0.6 in the study population.

A high density SNP genome scan was performed using a whole-genome sampling analysis (WGSA) approach [32] with the Affymetrix GeneChip Human Mapping 10K v2 Array (early access) comprising 10,660 SNP markers with an average heterozygosity in Caucasians of 38% and a mean spacing of 258 kb/0.36 cM (Affymetrix , NetAffx Annotation files, http://www.affymetrix.com). Mapping order and genetic distances of markers were obtained from Affymetrix, the genetic position of 86 markers was unavailable, 295 were X-linked, and 10,279 were from autosomes. Allele frequencies were estimated from 134 founders. The physical positions of the markers were aligned to human DNA sequence information available from NCBI/NIH (http://www.ncbi.nlm.nih.gov/mapview/maps.cgi).

### Statistical analysis.

In a first step, gender of participants was verified by investigating heterozygosity and hemizygosity, respectively, at X-linked markers. The relationships between individuals were confirmed by using Graphical Relationship Representation [47]. Genotypes incompatible with Mendelian inheritance were identified with PedCheck [48] and removed in members of the respective families. Unlikely genotypes, e.g., double recombinants, were investigated with Merlin [49], and apparent errors were resolved by deleting the respective genotypes of all family members. The genotypes of two participants showing <80% called genotypes were completely removed from the data. In a second step, SNP markers were excluded if they showed either (i) a deviation from Hardy-Weinberg equilibrium in founders at the nominal *p* < 0.001 test level, (ii) a genotype calling fraction of <80%, or (iii) a heterozygosity of <5%.

A quantitative trait locus autosomal linkage analysis was performed using the model-free nonparametric linkage method (NPL) [50] and the Haseman-Elston method (HE) [51], assuming an additive genetic model. In order to allow the simultaneous analysis of all SNP loci on a given chromosome in a multipoint approach, we adapted GENEHUNTER [52] to a 64 bit version [53]. Asymptotic *z* scores from NPL and LOD scores from HE are reported. Additionally, empirical *p*-values were determined using 100,000 replicates on the basis of the mean information content in the multipoint SNP analysis, which was 0.976 (SD ± 0.029, range 0.510–1.000). Specifically, a single marker was simulated with 20 equally frequent alleles, thus yielding a heterozygosity of 0.95. For the simulation, family structures and phenotypes were left unchanged. Details on this Monte-Carlo approach can be found, e.g., in [54]. A linkage signal of *p* < 10^−4^ in either NPL or HE was considered significant [55]. In addition, we describe two linkage signals which were below the threshold of significance, one signal of *p* < 5·10^−4^ and another signal of *p* < 10^−3^ obtained at the same genomic region with two largely independent phenotypes. We calculated locus-specific heritabilities of additive effects using the regression approach of Sham and colleagues [56]. Applying this method, we estimated the mean and variance from the data and fixed the heritability to 0.5. Decreasing the overall heritability led to moderate increases of the locus specific heritability. Candidate genes were defined as genes relevant to red blood cell structure, red blood cell metabolism or the inflammatory response.

## Abbreviations

HE: Haseman-Elston multipoint linkage analyses
LOD: logarithm of odds
NPL: nonparametric linkage analysis
PCV: packed blood-cell volume
